# Chandipura Viral Encephalitis: A Brief Review

**DOI:** 10.2174/1874357901812010044

**Published:** 2018-08-31

**Authors:** Gajanan N. Sapkal, Pradeep M. Sawant, Devendra T. Mourya

**Affiliations:** National Institute of Virology, 20-A, Dr. Ambedkar Road, Pune 411001, India

**Keywords:** Chandipura virus, Pediatric sporadic encephalitis, Sero-survey and diagnosis, Entomological surveillances, Vectors, Sandflies

## Abstract

**Introduction:**

In recent years, the Chandipura virus (CHPV) has emerged as an encephalitic pathogen and found associated with a number of outbreaks in different parts of India. Children under 15 years of age are most susceptible to natural infection. CHPV is emerging as a significant encephalitis, causing virus in the Indian subcontinent. Severe outbreaks caused by the virus have been reported from several parts of India.

**Expalanation:**

In the recent past, the noticeable association of CHPV with pediatric sporadic encephalitis cases as well as a number of outbreaks in Andhra Pradesh (2004, 2005, 2007 and 2008), Gujarat in (2005, 2009-12) and Vidarbha region of Maharashtra (2007, 2009-12) have been documented. Prevalence and seasonal activity of the virus in these regions are established by NIV through outbreak investigations, sero-survey and diagnosis of the referred clinical specimens. Recently CHPV has been isolated from pools of sand flies collected during outbreak investigations in Vidarbha region of Maharashtra. Since its discovery from India and above-mentioned activity of CHPV, it was suspected to be restricted only to India.

**Conclusion:**

However, CHPV has also been isolated from human cases during 1971-72 in Nigeria, and hedgehogs (*Atelerix spiculus*) during entomological surveillance in Senegal, Africa (1990-96) and recently referred samples from Bhutan and Nepal and from wild toque macaques (*Macaca sinica*) at Polonnaruwa, Sri Lanka during 1993 suggest its circulation in many tropical countries. Based on the limited study on vector related report, it appears that sandflies may be the principle vector.

## 1 INTRODUCTION

Chandipura virus (CHPV) was discovered during an acute febrile outbreak in Nagpur, Maharashtra state, India from two febrile cases [1965] [1, 2]. It belongs to the genus Vesiculovirus, family *Rhabdoviridae*. This virus has single-stranded RNA genome with negative polarity and size about 11 kilobases. Five structural proteins are coded by genome: the nucleocapsid protein (N), the phosphoprotein (P), the matrix protein (M), the glycoprotein (G) and large structural protein (L). These are produced in the form of five monocistronic mRNAs [3]. The available information suggests that sandflies are the vectors for this virus while antibodies against this have been detected in a wide range of vertebrate animals [4]. Cells of insect origin and vertebrate animals were found to be susceptible to virus replication. Interestingly, one of the bat cell-line that was refractory to replication of many Alpha- and flaviviruses was found susceptible to CHPV [5]. Similarly, the rate of replication of CHPV in the susceptible cell lines has been recorded extremely high thus within 24-48 hours, a complete monolayer of the cell sheet gets destroyed [6]. General clinical features include high-grade fever of short duration, vomiting, altered sensorium, generalized convulsions and decerabrate posture leading to grade 4 coma, acute encephalitis-encephalopathy and death within a few to 48 hours of hospitalization [7]. The available epidemiological data suggest that this disease mostly occurs in sporadic forms; however, has potential to cause outbreaks. This disease is not in a routine laboratory screening for acute encephalitis syndrome [AES]. Therefore, during any AES syndrome, the role of this virus is not understood. Besides this, there have been many queries about CHPV: is sandflies the only vector? What is the natural cycle? Do small mammal, domestic animals have any role and since the transovarial transmission of virus in sandflies may not be enough for its maintenance in nature. Understanding the natural cycle can help in interventions and prediction off or breaks. This article reviews research activities and further developments occurred during the last 10 years, which are necessary to understand CHPV as a growing concern in India, its epidemiology, diagnosis, vaccinology and biology of the virus.

## 2 DISTRIBUTION OF VIRUS

Till date, the presence of this virus is recorded from Indian subcontinent [India, Bhutan and Nepal], Sri Lanka, and Africa (Nigeria, Senegal) [8, 9]. Hence, it is speculated that CHPV may be present in other parts of the country. Although, this was first time identified in India during 1965 but the retrospective serological studies indicate exposure of the human population as early as during 1957–58 [1]. Earlier reports suggest that the association of CHPV with a few undiagnosed outbreaks occurred in 1954 in Bihar [10]. However, CHPV was isolated from sera collected from clinically confirmed encephalitis cases in 1983 from Raipur [11] and the Warangal district of Andhra Pradesh (1997 and 2002) [12] suggesting its wide circulation in the country. However, CHPV re-emerged during 2003 in the form of an encephalitis outbreak affecting 11 districts of Andhra Pradesh with a high fatality ratio of about 56% [13]. CHP encephalitis (CHPE) outbreak was simultaneously documented in 15 districts of Maharashtra during the same time [13, 14]. During the subsequent year (2005), CHPV outbreak with 70% case fatality rate in the pediatric population of Vadodara district of Gujarat was documented [14]. It has been associated with a number of encephalitis epidemics in different states of India viz. Andhra Pradesh in 2003 and 2007, Gujarat in 2004, Maharashtra in 2007 and 2009, and Odisha in 2015 [15, 16]. The CHPV has also been isolated in Nigeria from hedgehogs and in Sri Lanka from macaques [17] (Table **1**).

## 3 CASE DEFINITION

Fever (100%), convulsion (76.3%), altered sensorium (34.2%), headache (23.7%), vomiting (44.7%) and diarrhea (23.7%).

## 4 NATURAL CYCLE

Earlier studies revealed that CHPV is predominantly circulating in the central part of India. The presence of anti-CHPV neutralizing antibodies in the blood collected from pigs, buffalos, cattle, goats and sheep suggests continuous circulation of the virus in this region [18]. This demonstrates exposure of the domestic animals to CHPV.

It is interesting to note that earlier serological investigation of CHPV activity suspected in the areas of Andhra Pradesh, Maharashtra (Nagpur and Beed districts) and Karnataka (Bangalore) did not show anti-CHPV IgM antibodies in the 191 human sera. This is suggestive of sporadic nature of this virus. The anti-CHPV neutralizing antibodies have also been detected in the sera collected from frog (2/33), lizard (2/14) and Rodents (26/32). This indicates a probable role of these or such animals in maintaining the virus in nature [19]. However, this virus has capabilities to affect larger population and appear in outbreak form. Serological investigations of CHPV in Andhra Pradesh State demonstrated high-level exposure of the pediatric population as anti-CHPV neutralizing antibodies detected in about 81% (237/291) human sera [20]. The anti-CHPV seroprevalence was higher in the age group of >15 years in 33 (16 affected and 17 unaffected) localities of 6 districts in Maharashtra state. Anti-CHPV IgM antibodies were detected in 5.5% (30) sera and anti-CHPV neutralizing antibodies were detected in 15.1% (82) sera collected from pediatric population. As the laboratory data in mice indicates that infants are highly susceptible thus CFR is high in this pediatric age group, however, adults develop IgM antibodies. Besides studies have been conducted for understanding the role of bats in a natural cycle maintenance cycle.

## 5 DISEASE PATHOGENESIS

### 5.1 CHPV Infection in Laboratory Rodents (Mice & Rats)

Studies have shown that 16 days old mice when inoculated subcutaneous with CHPV, 5th-day post infection hind limb weakness was observed that continued to 7-8 PID and then mice recovered. However histological investigations showed no gross changes in any of the organs. With CHPV infection in the infant mice, frank sickness was observed with ataxia, hyperesthesia, convulsions, quadriplegia and death. Interestingly marked histological changes were observed only in the brain and spinal cord in gradation of the post-infection period. Earlier studies have shown that rats of two weeks age could be suitable animals for studying the pathogenesis, host-virus interaction, and drug development etc. for CHPV. Degeneration of neurons, Antigen detection in cytoplasm of neurons and chromatolysis of neurons as well as localization of antigen in Purkinje cells and choroid have been recorded [21]. Neuropathogenesis of virus has been established but its route of entry to the central nervous system (CNS) and mechanism of neuronal death is unknown. One way to enter the nervous system is by retrograde movement from peripheral nerves or olfactory nerves and the other one is through damaged blood-brain barrier by cytokines and chemokines produced in response to peripheral infection. After entering neurons, it triggers cellular stress factors and release of reactive oxygen species which initiate neuronal death [22]. Recent evidence shows that the virus induces death by triggering death domain or microglial activation [23].

### 5.2 Genetic Characterization and Phylogenetic Analysis of CHPV

Till date, a total of 8 complete genomes of CHPV isolated in India are available and 2 CHPV isolates have been documented in African continent [24]. The phylogenetic analyses of the whole genome sequences indicate stability of the virus isolates obtained during the last 47 years (1965-2012). The percent nucleotide divergence of the CHPV whole genomes isolated in India varied from 3.54–3.71 with respect to the prototype isolate of 1965. The comparison with full genome sequences of African continent indicates higher genetic divergence of 5-6% from Indian isolates indicating that these independently emerged in different continents and are independently evolving. These observations indicate that CHPV is independently evolving with time.

### 5.3 Bioinformatics Approach to Identify the Markers for Pathogenesis

Search of possible hotspots in complete genomic sequence of CHPV was compared with other members of Rhabdoviruses that may be responsible for pathogenesis. This virus is in the cluster with the Isfahan virus, however, maintains several functional motifs of other Rhabdoviruses. There is difference with the prototype Vesiculovirus in flanking sequences of the M protein. This is crucial for interaction with host proteins. Several mutations in G protein have been mapped onto probable antigenic sites. Mutations in N protein mapped have been shown crucial for N-N interaction and a putative T-cell epitope. A mutation in the Casein kinase II phosphorylation site in P protein may attribute to increased rates of phosphorylation [25]. Further protein-protein interaction between host and virus proteins is in progress to reveal ways by which the virus manipulates the biological pathways of host in its favour and evades immune system [26].

### 5.4 Immunological Markers

The susceptibility of mice and humans to CHPV infection is age-dependent. Experimental infection in mice secretes significant amounts of pro-inflammatory cytokines. Monocytes and B cells support active replication of CHPV. An elevated level of cytokines and chemokines observed in monocytes may help in predicting the pathogenicity of CHPV and possible entry into the central nervous system [27].

**Fig. (1):**
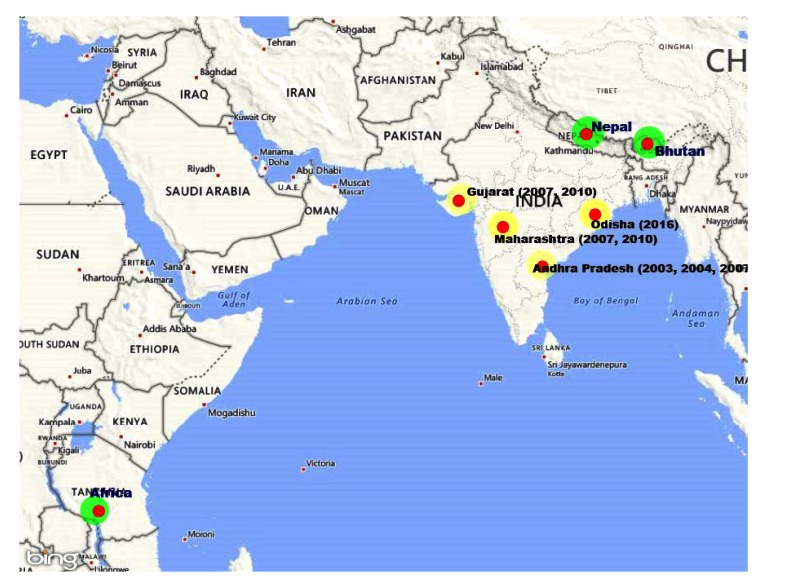
Recent distribution of Chandipura virus.

Children who recover from natural infection with the virus show significant amounts of TNF-α production, suggesting that innate immunity plays a major role in response to CHPV. TLR are key host molecules involved in innate immune responses in infections. CHPV infection activates TLR4, which leads to the secretion of pro-inflammatory cytokines and nitric oxide (NO). Despite activation of the innate immune system, mortality was observed in young mice. Partial protection in TLR4 mutant mice and NO inhibitor-treated wild-type mice indicated that TLR4 and NO contributes to disease pathogenesis [28, 29]. IL-2 was detected in most of the early acute cases, this probably is associated with recovery [30] (Fig. **1**).

During CHPV infection in mice, drastic reduction in CD4+, CD8 + and CD19 + cell was reported. Depletion of lymphocytes in spleen suggested that the reduction may be due to the regulatory mechanism of immune system to prevent the bystander host tissue injury.

The role of regulatory cells in homeostasis with regard to CD4+T regulatory cells from the infected mice suggests induction of CD4+T regulatory cells and expression of PD-1 in infected mice may be one of the mechanisms by which the immune system controls the activated lymphocytes and maintains homeostasis [31]. It was also reported that microglial activation might be one of the triggering factors for the neuronal apoptosis in CHPV infection [23].

### 5.5 Approaches to Vaccine Developments

Vero cell-based inactivated vaccine candidate against CHPV elicited efficient protection after two doses upon challenge with live virus (100PFU) in mice. Antibody titer after the third dose ranged between 1:80 and 1:320. Mice, which demonstrated neutralizing antibody titer above 1:20, survived live virus challenge through even intra cranial route [32]. In another approach, a candidate vaccine employing recombinant CHPV Glycoprotein gene (G-gene) using Baculovirus expression system showed intracerebral challenge to the immunized mice with 100 LD50 of the homologous strain with 90% protection [33].

### 5.6 Laboratory Transmission Experiments

Several studies demonstrated the venereal transmission of arboviruses by its arthropod vectors that might serve as one of the mechanisms for horizontal transmission. Lab experiments documented vertical and venereal transmission of CHPV in *Aedes aegypti*. The minimum infection rate among the progeny of infected females was documented to be 1.2%. The venereal infection rate of CHPV among inseminated females was 32.7%. The study indicated the possible occurrence of vertical and venereal transmission of CHPV in insect vectors. Experiments conducted on *Phlebotomus papatasi* to determine the possible role of males in maintaining or sustaining the CHPV activity in nature indicated that infected males are capable of passing on the virus to female sand flies while mating. The infection rate was found to be 12.5% in uninfected females when mated with infected males. The occurrence of venereal transmission of CHPV may contribute to the epidemiology and in the natural cycle of CHPV. In India, *P. argentipes* is one of the predominant sand fly species found in many CHPV endemic areas and 65% of the lab-grown *P. argentipes* were susceptible to CHPV infection by the oral route. Transmission experiments were also carried out by intra-thoracic inoculation because of re-feeding problems with this species. After incubation for 24 hours, efficient transmission of CHPV to mice was observed. The estimated minimum transmission rate among the inoculated flies was 32%. CHPV in sand flies as well as in mice, was detected and confirmed by immunofluorescent antibody assay and RT-PCR assays. The susceptibility of *P. argentipes* to CHPV and its potential to transmit the virus by bite might contribute towards the natural transmission of CHPV [34, 35].

## 6 CHANDIPURA DIAGNOSIS

### 6.1 Molecular Diagnostic Assays for CHPV

CHPV causes acute encephalitis in pediatric population under the age of 15 years. The critical feature of CHPE is sudden onset of the clinical symptoms including neurological complications (within 24-30 hrs.) and high fatality rate. Due to the short duration between the onset of clinical feature and neurological illness, serological diagnostsis is not useful. The virus has been detected in CSF as well as sera collected from the patients in the acute phase of illness using CHPV specific one-step RT-PCR assay that detects 10-100 pfu / ml of the virus in human clinical specimens. Real-time one-step RT-PCR indicates linear relationship for a wide range of viral RNA 10^2^-10^10^. When RNA from other viruses or healthy individual was used, specificity was found to be 100% [36].

### 6.2 Serological Diagnosis Assay for CHPV

CHPV specific IgM capture ELISA with specific polyclonal antibodies shows polyclonal antibodies masking the specificity of the assay to be used for the detection of anti-CHPV IgM antibodies in the patient’s CSF and sera. Monoclonal antibodies were generated and replaced in anti-CHPV IgM ELISA to increase the sensitivity, specificity and rapidity of the assay [37]. Plaque reduction neutralization test (PRNT) is considered as ‘gold standard’ to detect neutralizing antibodies against Chandipura virus. However, the test is cumbersome to perform, time intensive and reading is subjective. Recently developed micro-neutralization ELISA (MN ELISA) detects neutralizing antibodies against CHPV with readouts in the form of optical density and shorter turnaround time. This test may serve as an alternative to conventional assay in serosurveillance and vaccine studies [38].

### 6.3 Future Directions of CHPV Research in India

CHPV activity was detected in India in a few selected geographic regions of the country. However, further investigations in the country and in other geographic regions are needed. The natural cycle of CHPV is still not clear but the demonstration of anti-CHPV antibodies in rodents, cattle, sheep, goat, pigs, frog, hedgehogs, lizard and rodents etc. indicates the wider range of the amplification/maintenance hosts explored by the virus in nature [39]. Further studies are necessary to unravel the animal species that are infected by the virus in nature. The actual nature of the transmission vector in nature is resolved but frequent isolation of CHPV from sand flies may relate them as the principal vector of transmission in nature.

Evolutionary studies on CHPV indicate substantial genomic and antigenic stability of the virus during the last 47 years. Hence, it is possible to obtain protection against all the circulating strains with a vaccine developed against any one of the strains. The efforts are needed for CHPV vaccine development research for protective vaccine candidate. Alternative approaches should also be considered while planning for complete cure of the problem by adopting novel technologies boosting production and safeguarding health of humans and animals, through the use of immunomodulation and immunomodulatory agents on health with some bioactive principles, modes of action and potent biomedical applications and the innate immune receptors with ingenious anti-viral roles [40-42]. However, further efforts are necessary to use it for the susceptible population, hence, the development of ELISA for serological investigations is needed.

## Figures and Tables

**Table 1:** Chandipura activity globally.

1965	First detected and isolated virus from two febrile cases in Nagpur, Maharashtra state, India [1, 2]
1997 & 2002	Warangal district, Andhra Pradesh investigations suggested its wide circulation in the country
2003	Andhra Pradesh 11 districts encephalitis outbreak affecting with CFR 56%
2003	Andhra Pradesh, India isolates.
2004	Vadodara district of Gujarat CHPV outbreak with 70% CFR in the pediatric population
2004	Outbreak in Gujarat State, India, CFR 78.3% among children, virus isolated
2004-05	Anti-CHPV neutralizing antibodies (NAbs) in 65.3% sera CHPV was isolated
2005-2006	Andhra Pradesh Hospital-based surveillance, about 54.4% CFR
2007-2008	Investigation in Nagpur Maharashtra, CDR 43.6%, viral RNA detected in sandflies from affected areas.
2008	CHPV activity in Maharashtra RNA was detected in two pools of sand flies
2009	Andhra Pradesh investigations on hospitalized encephalitis cases CHPV infection in 8/10 by RT-PCR and while only one of the 132 contact sera collected in Hyderabad region 1 showed anti CHPV IgM abs.
2010-11	Panchamahal district Gujarat, from fever cases anti-CHPV IgM antibodies detected in 10.56% [n=587] sera.
2012	Encephalitis cases from Maharashtra and Gujarat states anti-CHPV IgM ELISA and RT-PCR confirmed 4.7% [n=130] sera
Till date	Presence of virus recorded from Indian subcontinent [India, Bhutan, Sri Lanka and Nepal] and from African continent Nigeria, Senegal from hedgehogs.
